# Vectorcardiography-derived index allows a robust quantification of ventricular electrical synchrony

**DOI:** 10.1038/s41598-022-14000-8

**Published:** 2022-06-15

**Authors:** Juan M. F. Fernández, Damián N. Spagnuolo, María T. Politi, Iván A. Tello Santacruz, Miguel Schiavone, César Cáceres Monié, Horacio A. Avaca, Osvaldo Chara

**Affiliations:** 1 Systems Biology Group (SysBio), Institute of Physics of Liquids and Biological Systems (IFLySIB), National Scientific and Technical Research Council (CONICET), University of La Plata, La Plata, Argentina; 2 Laboratory of Biomembranes, Institute of Physiology and Biophysics Bernardo Houssay (IFIBIO Houssay), School of Medicine, University of Buenos Aires, Buenos Aires, Argentina; 3 Cardiology Department, British Hospital of Buenos Aires, Buenos Aires, Argentina; 4 Center for Information Services and High Performance Computing, Technische Universität Dresden, Dresden, Germany; 5 Instituto de Tecnología, Universidad Argentina de La Empresa (UADE), Buenos Aires, Argentina

**Keywords:** Cardiac device therapy, Heart failure, Translational research

## Abstract

Alteration of muscle activation sequence is a key mechanism in heart failure with reduced ejection fraction. Successful cardiac resynchronization therapy (CRT), which has become standard therapy in these patients, is limited by the lack of precise dyssynchrony quantification. We implemented a computational pipeline that allows assessment of ventricular dyssynchrony by vectorcardiogram reconstruction from the patient’s electrocardiogram. We defined a ventricular dyssynchrony index as the distance between the voltage and speed time integrals of an individual observation and the linear fit of these variables obtained from a healthy population. The pipeline was tested in a 1914-patient population. The dyssynchrony index showed minimum values in heathy controls and maximum values in patients with left bundle branch block (LBBB) or with a pacemaker (PM). We established a critical dyssynchrony index value that discriminates electrical dyssynchronous patterns (LBBB and PM) from ventricular synchrony. In 10 patients with PM or CRT devices, dyssynchrony indexes above the critical value were associated with high time to peak strain standard deviation, an echocardiographic measure of mechanical dyssynchrony. Our index proves to be a promising tool to evaluate ventricular activation dyssynchrony, potentially enhancing the selection of candidates for CRT, device configuration during implantation, and post-implant optimization.

## Introduction

Chronic heart failure is one of the leading health and economical burdens worldwide, yielding high morbidity and mortality rates^1^. During its end-stages, this pathology is characterized by altered ventricular electrical activation and ventricular arrhythmia. One of the therapeutic approaches for end-stage heart failure consists in the implantation of cardiac resynchronization therapy (CRT) devices in a selected group of patients to improve the heart’s mechanical efficiency. Specifically, CRT is strongly recommended in patients with a left bundle branch block (LBBB) and reduced left ventricle (LV) ejection fraction with persistent heart failure symptoms despite optimal medical treatment. Also, CRT can be considered for other patients based on weaker recommendations^2,3^. When successful, this artificial stimulation from specific heart locations improves both mechanical cardiac efficiency and patients’ clinical condition. However, individual response to CRT is hard to predict: currently, up to 30% of all patients treated with CRT do not show significant clinical improvement^4^. Additionally, guideline recommendations are also believed to exclude other patients who could probably benefit from CRT, since the QRS morphology is not the only determinant of dyssynchrony^5^. An important source of this classification problem is the lack of a precise method for quantifying ventricular synchrony in order to identify patients with more dyssynchronous ventricular activations and, therefore, with higher chances to benefit from resynchronization. Thus, there is a clear clinical need for an accurate quantification of ventricular synchrony for predicting the therapeutic response to CRT. Additionally, this quantification could also be useful for guiding cardiac electrophysiologists in selecting the best configuration for each patient during or after CRT device placement.

Recently, attention has been drawn towards vectorcardiogram (VCG) as a tool for cardiac electrical synchrony assessment in CRT candidates. Particular emphasis has been placed on attempting to predict CRT therapeutic response based on the quantification of VCG-derived QRS area (QRS_AREA_), which results from the numerical integration of the VCG vector magnitude over time (in this study, referred to as “voltage time integral”, VTI)^6,7^. Similarly, other related magnitudes such as QRS-T area and QRS_AREA_ in the Z dimension have also been proposed^8,9^^.^

Although the aforementioned quantities represent an improvement compared to the current electrocardiographic-based CRT candidate selection criteria, the complexity of the determinants of cardiac synchrony in general and the complexity of the determinants of QRS_AREA_ in particular open the possibility of further advances (see Discussion section). In this study, we propose a novel index to measure the ventricular electrical activation synchrony, implemented in a computational pipeline. After software validation using an online ECG database^10^, we applied our method to automatically analyse a local database of nearly 2000 ECGs. Finally, we investigated the link between our dyssynchrony index and mechanical heterogeneity in LV contraction by performing speckle tracking echocardiography during post implant follow-up in patients with pacemaker (PM) or CRT device.

## Results

### ECG-derived VCG analysis of typical conduction patterns

**Figure 1:**
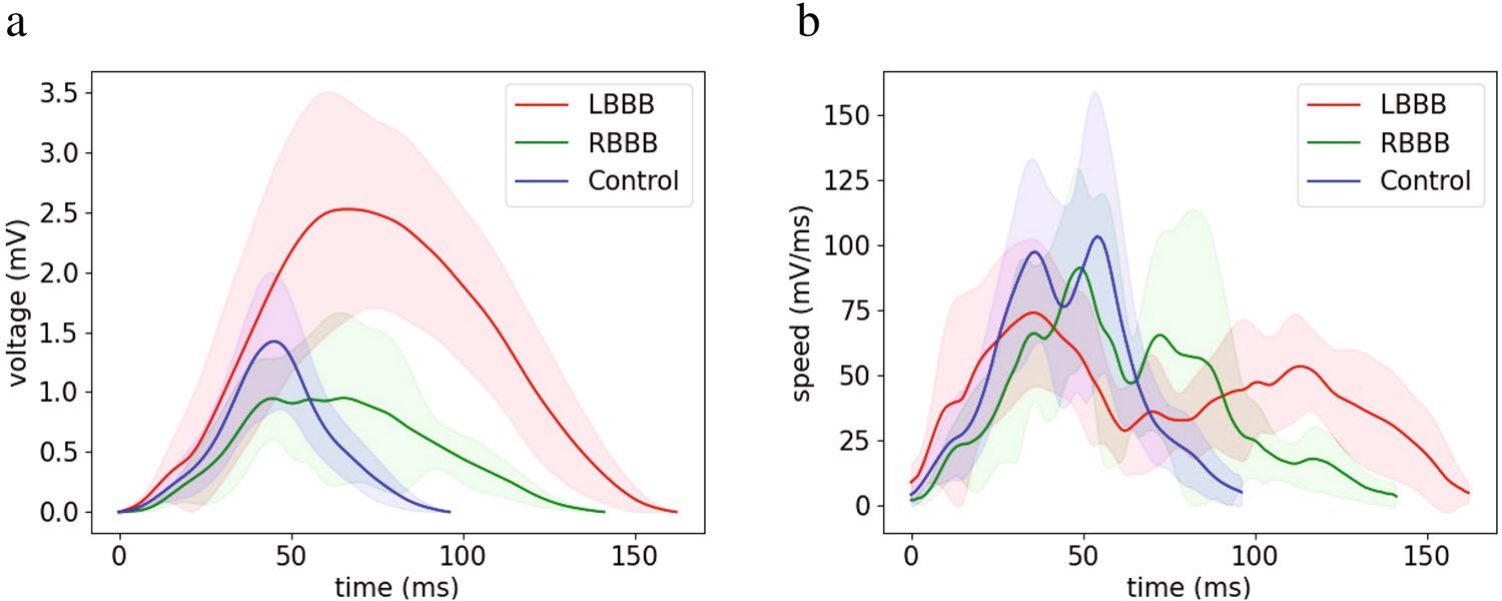
Population-wise kinetics of voltage and speed signals for left and right bundle branch block (LBBB and RBBB) compared with control patients. Signal lengths were normalized to the mean length to obtain the average signal for each condition. Shaded areas represent the standard deviation. (**a**) Voltage over time signals for control subjects (blue, n = 49), patients with right bundle branch block (RBBB, green, n = 9) or left bundle branch block (LBBB, red, n = 8) obtained from the PTB database. LBBB signals show higher voltage values and signal duration as compared with controls or RBBB. (**b**) Vector speed over time for the same three conditions. LBBB and RBBB signals show slower movement through the vector field as compared with controls. The figure was created using Python’s Matplotlib library (v.3.4.2.)^37^.

Through the use of Kors’ transformation matrix^11^ (see Methods section), we reconstructed the VCGs of 49 control individuals, 9 patients with RBBB and 8 patients with LBBB. ECG signals were obtained from the online PTB Diagnostic ECG database available on the Physionet website^10,12^. The trajectory of the vectors in the three-dimensional space is clearly altered in the VCG of patients with RBBB or LBBB as compared with controls (see representative examples in Fig. S1). Although our method can select a number of outputs in a variety of visualizations, in this study we decided to overcome the complexity of the 3D representation by focusing on two variables: voltage and speed. From the simple observation of the average curves, LBBB signals showed greater vector magnitudes with slower movements in the vector field as compared with controls, while RBBB signals showed intermediate values for vector magnitudes and speeds that were between LBBB and control values (Fig. 1a, b, see representative examples in Fig. S1).

### The VCG-derived speed time integral (STI) is proportional to the voltage time integral (VTI) in healthy controls

**Figure 2:**
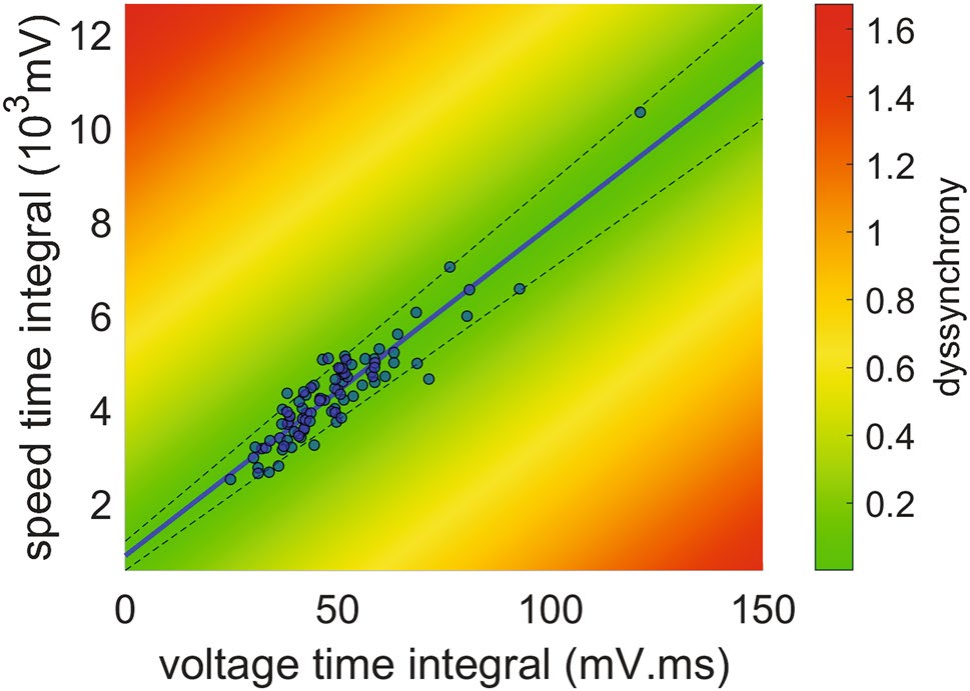
Heatmap showing the dyssynchrony index and the VCG-based time integral of voltage as well as speed from healthy individuals. Vector speed time integral (STI) plotted against vector voltage time integral (VTI) for 90 control individuals. The blue line is the linear fit of the data (r^2^ = 0.86, dotted lines: 95% confidence interval). The line’s slope represents the tissue characteristic time (70.28 ms^−1^). The colour bar highlights the dyssynchrony index as defined in the Methods section. The figure was created using MATLAB R2019b (https://www.mathworks.com/).

We extracted the VCGs of 41 local university students (22–48 year-old, healthy volunteers with no history of cardiovascular pathology) (US database). The voltage and speed kinetics of the vectors of ventricular depolarization from this database overlapped the signals of the control individuals from the online PTB database (Fig. S2a,b). In accordance, the VTI and STI of these controls from both samples did not differ significantly from one another (43.66 [40.56–56.82] mV ms vs. 49.34 mV ms [38.65–52.50] mV ms, p = 0.75 and 3920 [3476–5011] mV vs. 4324 [3853–4756] mV, p = 0.37 for the VTI and STI comparisons respectively, Fig. S2c,d). Considering these similarities, we pooled these observations together into one single group named “control population”, that contained the controls from the online PTB database and the local US database. In order to determine whether the VTI and STI were associated, we examined their behaviour within the control population. Indeed, these variables showed a coefficient of determination close to one (r^2^ = 0.86, p < 0.0001). On a scatter plot representing STI over VTI, the observations fitted neatly into a linear function (Fig. 2) with a slope (70.28 ms^−1^) that represents the inverse of the tissue’s characteristic time and describes the relation between these two variables in the population of individuals without intraventricular conduction abnormalities. We hypothesized that the observations that lay in the proximity of the control population linear fit would belong to patients without intraventricular conduction alterations, while those far away would belong to patients with altered electrical conduction during the ventricle depolarization. More specifically, we entertained the hypothesis that the further from the control population line, the higher the dyssynchrony. To perform a quantitative assessment of our hypothesis we defined the dyssynchrony index as the normalized nondimensional perpendicular distance between the VTI-STI coordinates of an individual observation and the linear fit of the control population (colour map in Fig. 2).

### The STI-VTI plots identify patients with alterations in the ventricular depolarization sequence

Patients with LBBB or RBBB from the online PTB database lay mostly farther away from the control population’s fitted line on the STI over VTI plot. The median dyssynchrony index of patients with RBBB was higher than controls, although no significant (0.059 [0.039–0.172] vs. 0.039 [0.017–0.064]; p = 0.08) (Fig. S3a,b,d). Patients with LBBB were even farther from the control line than patients with RBBB (Fig. S3c), possibly reflecting the higher level of dyssynchrony expected from this condition. As a consequence, patients with LBBB presented a significantly higher median dyssynchrony index as compared with controls (1.195 [0.777–1.626] vs. 0.039 [0.017–0.064]) and with patients with RBBB (1.195 [0.777–1.626] vs. 0.059 [0.039–0.172]) (p < 0.0001, for both comparisons vs. control population and vs. RBBB) (Fig. S3d), suggesting that our hypothesis holds true. This result may be explained at least partially by the fact that voltage is in part determined by ventricular mass, while spatial vector speed could be related to the propagation speed of the depolarization wave (see Discussion section)^13^.

Since the dyssynchrony index could distinguish groups from an online database with clear differences in their level of dyssynchrony, we decided to test the ability of this index to discriminate among different degrees of dyssynchrony within a more complex and heterogeneous population of patients who displayed more diverse electrical conduction patterns, including—but not limited to—RBBB and LBBB. In order to do this, we retrospectively explored the ECGs of 1914 patients from the British Hospital of Buenos Aires, who were evaluated for suspected or confirmed arterial hypertension between February and November 2019 (local BH database). We choose this population to expand the external validity of our results since it included a diverse group of patients with a wide range of comorbidities and clinical conditions and with varying types and degrees of conduction abnormalities. This population also included healthy individuals with suspected—but latter discarded—arterial hypertension.

As a first step, ECGs from the local BH database (n = 1914) were independently inspected by two experienced cardiologists and classified into one of eight groups, depending on the intraventricular conduction pattern (Normal Conduction (NC), Incomplete Right Bundle Branch Block (IRBBB), Left Anterior Fascicular Block (LAFB), RBBB, Left Posterior Fascicular Block (LPFB), RBBB + LAFB, Incomplete Left Bundle Branch Block (ILBBB), LBBB and PM). Both cardiologists classified the ECGs according to pre-established criteria^14^. Disagreements between the two observers were identified and reviewed (14% of total ECGs, mostly IRBBB/NC).

**Figure 3:**
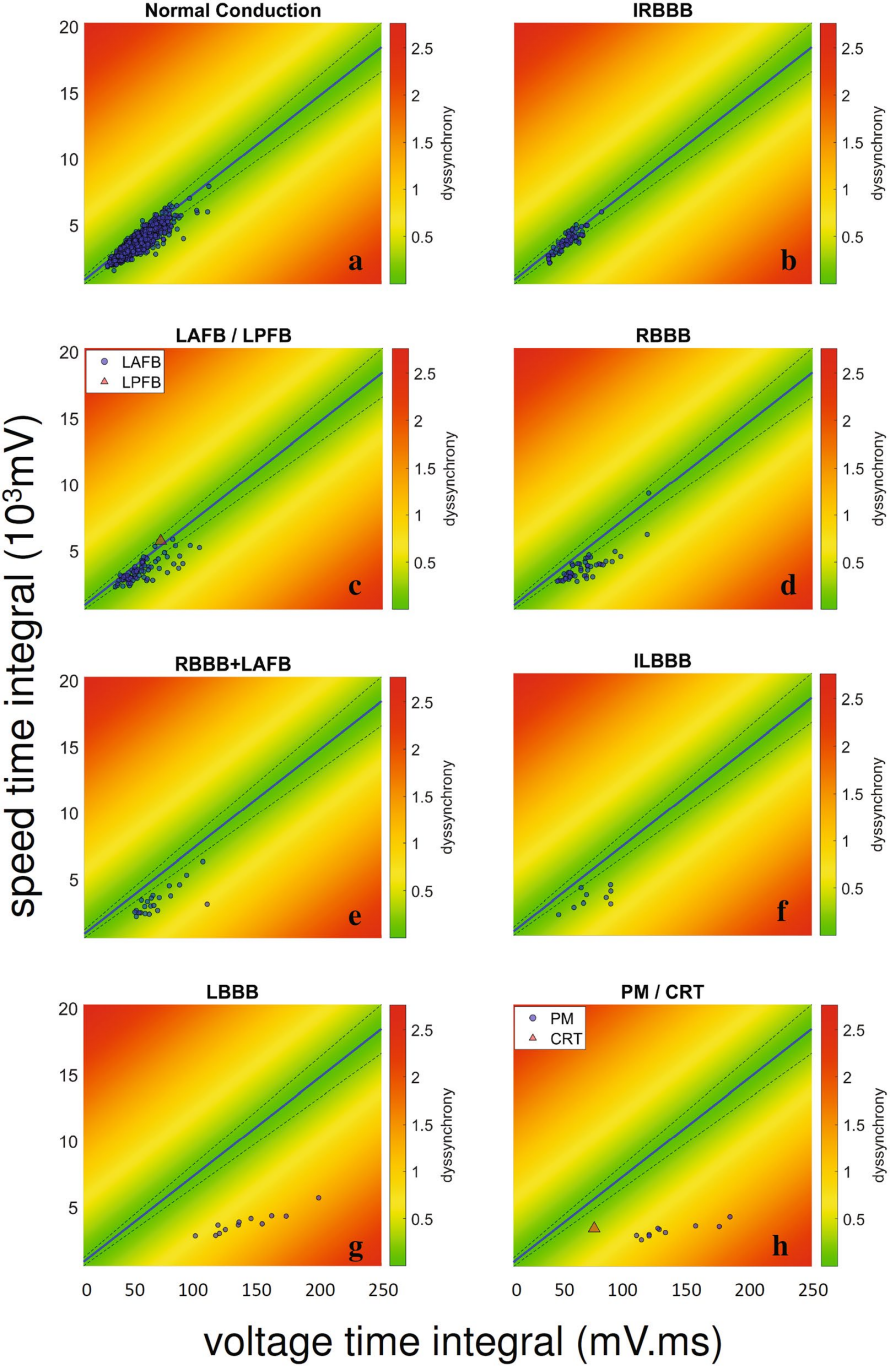
Heatmap showing the dyssynchrony index and the VCG-based time integral of voltage as well as speed for different conduction patterns in a heterogeneous population (local BH database). Each dot represents one patient, the blue line is the linear fit for the control population (Fig. 2) and dashed lines contain the 95% confidence interval (CI). Colorbar represent the value of the dyssynchrony index. Each patient was classified in its conduction pattern category by two cardiologists. Speed time integral (STI) *vs.* voltage time integral (VTI) plot for ECGs classified as normal conduction (**a**, n = 1672), incomplete right bundle branch block (IRBBB, **b**, n = 63), left anterior/left posterior fascicular flock (LAFB/LPFB, **c**, n = 78), complete right bundle branch block (RBBB, **d**, n = 47), right bundle branch block plus left anterior fascicular block (RBBB + LAFB, **e**, n = 22), incomplete left bundle branch block (ILBBB, **f**, n = 9), complete left bundle branch block (LBBB, **g**, n = 12) and pacemaker/cardiac resynchronization therapy (PM/CRT, **h**, n = 11). The figure was created using MATLAB R2019b (https://www.mathworks.com/).

The algorithm automatically analysed each of the 1914 ECGs. The results were then grouped according to the cardiologists’ classification. Figure 3 shows the dyssynchrony index of all patient from the local BH database, plotted along with the control population linear fit and 95% confidence intervals. Most patients from the local BH database labelled as “normal conduction” lay in the region closest to the control population fit (Fig. 3a). Not surprisingly, patients with IRBBB were also near the control population linear fit, since IRBBB is known to be a non-pathological conduction variant, which may not even modify the ventricular activation synchrony^15^. The overall behaviour of patients with LAFB, RBBB, RBBB + LAFB or ILBBB fell away from the control population line and presented varying dyssynchrony index values (Fig. 3c–f). The highest values where found in the LBBB and PM populations (Fig. 3g, h) with the remarkable exception of one patient with a CRT device (Fig. 3h, red triangle). To summarize, these results suggest that our dyssynchrony index reflects the magnitude of the electrical activation impairment generated by the conduction abnormality (Fig. S4).

To take our results a step further, we sought to formally define the limit between synchronous and dyssynchronous conduction patterns in order to increase the clinical applicability of our findings. We categorized the ECGs from all three databases (i.e., the online PTB database, the local US database, and the local BH database) into two groups: those with an electrical pattern that clearly conditioned dyssynchrony (patients with LBBB or PM) and those who’s electrical pattern could not be clearly categorized as dyssynchronous, at least not with a high level of certainty (NC, IRBBB, RBBB, RBBB + LAFB, ILBBB). Using a logistic regression model, we estimated the probability of being classified in each group based on the dyssynchrony index. Finally, we established the cut-off values that yielded the best discrimination performance among groups according to the highest Youden index^16^. The dyssynchrony index values with the best discrimination performance ranged from 0.519 to 0.657 with an average value of 0.588 (Fig. S5, see the Methods section, subsection “Statistical methods”).

### The dyssynchrony index identifies mechanically dyssynchronous patients

**Figure 4:**
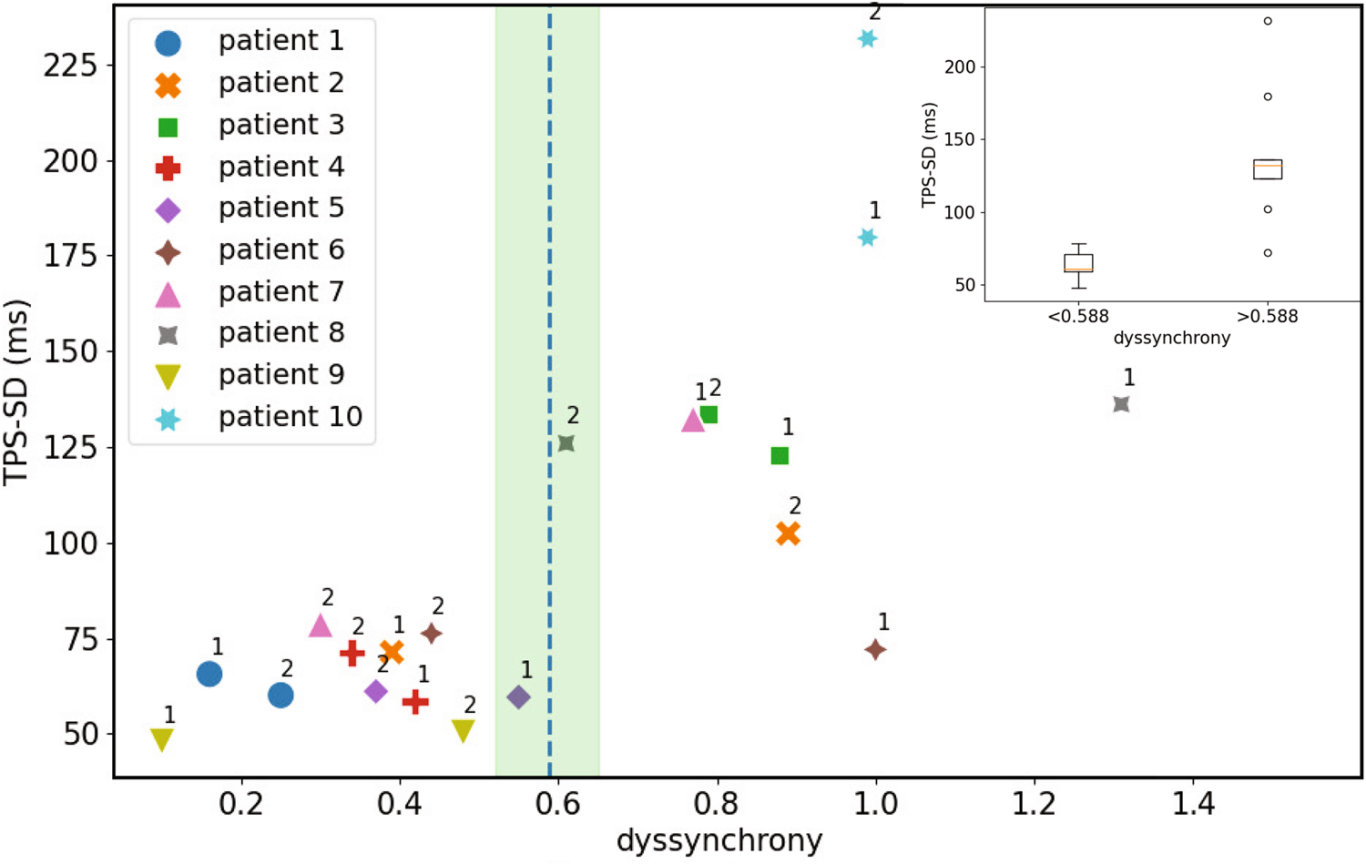
Simultaneous quantification of mechanical dyssynchrony and the dyssynchrony index. Standard deviation of the time to peak strain (TPS-SD) plotted against the dyssynchrony index. The marker styles represent different patients, 1 and 2 represent the ventricular stimulations denoted in Table 1. Green shaded area and blue vertical dashed line indicate, respectively, the range (0.519–0.657) and average (0.588) of the optimal cut-off values determined by using a logistic regression analysis (see the Methods section, subsection Statistical Methods and Fig. S5). Inset: Box plot showing TPS-SD for 10 patients (20 pacing configurations) grouped according to the average cut-off value for the dyssynchrony index (p = 0.00031). The figure was created using Python’s Matplotlib library (v.3.4.2.)^37^.

Since our results indicated that the dyssynchrony index was able to recognize alterations in the ventricular depolarization sequence, we wondered whether it could also identify mechanical dyssynchrony. To address this point, we obtained the echocardiographic images and ECGs (registered during the same office visit for device configuration optimization) from 10 patients with PM or CRT devices, and compared our ECG-derived dyssynchrony index with the echocardiographic time to peak strain standard deviation (TPS-SD) as a marker of mechanical dyssynchrony (see the Methods section, subsection “Mechanical synchrony assessment”)^17,18^. Two echocardiograms and their corresponding ECGs were recorded for each patient before and after device configuration optimization, according to the physicians’ personal expertise (see patient’s characteristics and pacing configurations in Table 1). Remarkably, almost all pacing configurations that presented a more homogeneous contraction (reflected in a low TPS-SD value) showed a dyssynchrony index below 0.588 (i.e., the average cut-off value with the best discrimination performance between LBBB/PM and non-LBBB/PM ECGs, Fig. 4 and Fig. S5). We found a statistically significant difference between TPS-SD values corresponding to a dyssynchrony index below 0.588 compared with those with a dyssynchrony index above this cut-off value (61.09 [58.98–71.22] ms vs. 132.23 [122.88–136.07] ms, p = 0.00031, Fig. 4 inset). Interestingly, this statistical significance was maintained even for the upper and lower limits of the range of dyssynchrony index values with best discrimination performance (0.519 and 0.657, p = 0.0019 and p = 0.00091 respectively), as described in the subsection “Statistical methods”. These results indicate that our dyssynchrony index is also a robust marker for mechanical dyssynchrony.

**Table 1:** Patients’ clinical data.

Patient ID	Male/Female	Age (y/o)	Cardiopathy	Device	Ventricular stimulation 1	Ventricular stimulation 2	Basal EF	AA Drugs	Other
1	F	63	AV block	His bundle PM (DDD)	Spontaneous (RBBB)	His pacing (AV time 120 ms)	Preserved	Sotalol	Mechanical aortic prosthesis
2	M	87	AV block Non-ischemic cardiomyopathy	His bundle PM (DDD)	Spontaneous (LBBB)	His pacing (AV time 120 ms)	Severe	Bisoprolol	
3	M	78	Ischemic cardiomyopathy	His bundle + RV PM (DDD)	RV	His-RV sequential pacing (80 ms delay)	Severe	Bisoprolol	CABG
4	M	54	Non-ischemic cardiomyopathy	CRT (CS cable)	LV-RV simultaneous	LV-RV (20 ms delay)	Severe	Bisoprolol	
5	M	64	Ischemic cardiomyopathy	His bundle PM (DDD)	Spontaneous (LBBB)	His-RV sequential pacing (80 ms delay)	Severe	Bisoprolol	
6	M	42	Non-ischemic cardiomyopathy	CRT (LV Epi. cable)	RV-LV sequential (80 ms delay)	LV-RV sequential (20 ms)	Normal (inferior limit)	Bisoprolol	
7	M	84	Ischemic cardiomyopathy	CRT (LV Epi. cable)	RV-LV sequential pacing (80 ms delay)	LV-RV sequential pacing (50 ms delay)	Moderate	Bisoprolol	AF (SR at the time of recording)
								Amiodarone	MVR
									CABG
8	M	70	Ischemic cardiomyopathy	CRT (CS cable)	RV pacing	RV-LV sequential pacing (40 ms)	Severe	Bisoprolol	CABG
								Digoxine	AF
9	M	81	AV block; Ischemic cardiomyopathy	His bundle PM (DDD)	Spontaneous (RBBB)	His pacing (AV time 120 ms)	Mild	Sotalol	MVR
									TVR
									AFL (ablation)
10	M	84	Ischemic cardiomyopathy	His bundle + RV PM (DDD)	RV pacing	RV-His sequential pacing (60 ms delay)	Severe	Bsioprolol	AF (SR at the time of recording)
								Amiodarone	
								Digoxine	

*AA* antiarrhythmic, *AF* atrial fibrillation, *AFL* atrial flutter, *AV* atrioventricular, *CABG* coronary artery bypass graft, *CRT* cardiac resynchronization therapy, *CS* coronary sinus, DDD refers to the pacing configuration in which there is as dual pacing, sensing, and inhibition, *EF* ejection fraction, categorized according to guidelines^19^, *Epi* epicardial, *LV* left ventricle, *LBBB* left bundle branch block, *MVR* mitral valve repair, *PM* pacemaker, *RV* right ventricle, *SR* sinus rhythm, *TVR* tricuspid valve repair.

## Discussion

Current heart failure and resynchronization guidelines clearly indicate the implantation of a CRT device in patients with LV ejection fraction below 35%, sinus rhythm, New York Heart Association functional class II, III, or ambulatory IV despite optimal medical treatment, and LBBB with a QRS width over 150 ms^2,3^. Although great efforts have been made to refine CRT indication criteria, around 30% of patients who receive this treatment obtain no benefit, leading to inefficient resource allocation and unnecessary patient risk^4^. On the other hand, guideline recommendations may also exclude patients who could be potentially benefit from CRT treatment, for example certain subgroups of non-LBBB patients^5,20,21^. Hence, the two main challenges CRT is currently facing are: how to accurately identify patients who will respond positively to treatment, and how to measure the improvement in synchrony produced by CRT in order to setup an optimal stimulation sequence. Both of these major issues are rooted in the lack of an accurate method for quantifying synchrony. Precisely, in this study we conceived, computationally implemented, validated, and tested a pipeline to extract an index of dyssynchrony from ECG-derived VCGs.

Currently, the best-known ventricular synchrony estimator is the QRS width. Along with QRS morphology, this is the only electrical synchrony estimator to be included in guideline recommendations for the indication of CRT in patients with heart failure^2,3^. Though a dyssynchronous ventricular activation is characterized by an abnormally prolonged ventricular depolarization time, it is now clear that this is not the only factor at stake, and perhaps not even the most important one. Given the aforementioned high prevalence of CRT non-responders, new VCG-derived estimators of dyssynchrony have been proposed, such as the area under the curve of the ventricular depolarization vector magnitude versus time plot (referred to in the literature as “QRS_AREA_” and in this study as “voltage time integral”, VTI) or the related magnitude QRST integral^6–8^. The idea that voltage-derived variables, such as QRS_AREA_, should outperform QRS width in quantifying dyssynchrony and predicting CRT response is based on multiple assumptions, some biological and others methodological. The authors that developed and proposed these variables argued that the higher the voltage (e.g., the larger the QRS_AREA_) the greater the amount of tissue with a delayed activation that results in unopposed electrical forces towards the end of ventricular depolarization^22^. In addition, voltage magnitude depends on cell viability, since cells from scar tissue do not develop action potentials and, therefore, QRS_AREA_ could be inversely related to the amount of fibrotic tissue (which is not possible to resynchronize). QRS_AREA_ is also sensitive to cell-to-cell electrical coupling, independently of the presence of conduction blocks^23^. Finally, while QRS width and classification widely depend on the specific criteria used and on the observer’s impression, the area under the curve of the voltage versus time plot relies much less on these variables. Together, these characteristics of QRS_AREA_ make it a very good indicator of electrical synchrony and this magnitude has been proposed as a synchrony estimator for predicting CRT response with a performance better than QRS width alone, and just as good as the combination of QRS width and LBBB morphology^7^. However, the use of QRS_AREA_ and similar estimators entails pitfalls that need to be addressed. First, there is no consensus on the QRS_AREA_ cut-off value with best performance for quantifying dyssynchrony and predicting CRT response^6,7,22,24,25^. Second, there is also no clear agreement on which QRS_AREA_ to measure, since most authors use 3D QRS_AREA_ while others propose different quantities, such as QRS_AREA_ over the Z axis or QRST integral^8,9,26^. Third, the use of the Kors-derived QRS_AREA_ for predicting CRT response is largely based on small and medium-sized retrospective studies^7,24,25^. In these studies, cardiac mortality, overall mortality or combined end-points (LV assist device implantation, cardiac transplantation, or all-cause mortality) are evaluated as primary and secondary outcomes. QRS_AREA_, analysed alternatively as a continuous or dichotomous variable using several different cut-off values (95 mV ms, 102 mV ms or 109 mV ms), suggested a modest improvement (95 mV ms: HR 1.65 [1.25–2.18]; 102 mV ms: HR 0.33 [0.19–0.57]; 109 mV ms: HR 0.49 [0.41–0.59]) compared to QRS morphology and QRS duration, which were usually evaluated separately. Only one of these studies analysed the additional predictive value of QRS_AREA_ on top of class I indication (with LBBB and QRS duration ≥ 150 ms), showing a moderate performance (HR 0.54 [0.41–0.70])^7^. Therefore, despite the great value of these studies, QRS_AREA_ is far from being an ultimate solution to synchrony assessment, a fact that is reflected in its absence from clinical guidelines^2,3,27^.

Finally, although QRS_AREA_ clearly contains more information on ventricular depolarization than QRS width, it lacks information on the temporal dynamics of ventricular depolarization. For instance, a narrow QRS with high voltage can have the same area under the curve as a wide QRS with low voltage. Hence, the advantages of using a magnitude such as QRS_AREA_ could be potentiated by incorporating a variable that accounts for the time dynamics of ventricular depolarization. Our results indicate that this variable could be the vector speed.

Given the dependence of signal voltage on conducting tissue mass^28^, and our observation of a linear relationship between VTI and STI, we hypothesize that in physiological conditions tissue conduction is optimized to depolarize the entire ventricular mass in a very narrow time frame. Specifically, we speculate that this optimization probably involves the anatomical and electrophysiological properties of the heart’s conduction system, together with myocyte membrane channels, cytoplasm conductivity and intercellular gap junctions. We propose that for a given voltage there is a narrow range of optimal tissue conduction speeds, and that alterations in this relationship could indicate an electrophysiological impairment, which may lead to dyssynchrony and, ultimately, to mechanical impairment. In our study, the analysis of circa 100 control individuals allowed us to find a positive linear correlation between the ventricular depolarization STI and VTI from which we were able to extract the slope characterizing normal ventricular conduction. Following a similar approach, vector magnitude and speed from different conduction patterns were previously described in the 1960s in dogs and humans^13,29^, although no further attention was given to these curves, and no relationship between these variables was determined. To our knowledge, this is the first time that both vector magnitude and speed are included in the analysis of ventricular activation synchrony. Although the vector’s speed does not represent the speed of the depolarization wave itself, these are probably related since the vector’s speed derives from changes in the spatial angles and magnitudes of consecutive vectors^13^. Therefore, the inclusion of this variable in the analysis of ventricular activation synchrony could contribute to overcome the limitations of previous methods by taking into account tissue conduction properties, at least indirectly. Since in our study normal wavefront propagation is characterized by a linear relationship between voltage and speed, the quantification of the deviation from this line provided by our dyssynchrony index is likely to be independent of non synchrony-related variables such as the amount of cardiac mass and the electrical properties of non-cardiac intrathoracic tissue (i.e. volumetric conductor properties).

The voltage-speed time integral plot proposed in this study could be interpreted as a phase space, which is a popular representation for dynamic systems in Physics. In this space, there is a region near the control line where the probability of finding ventricular dyssynchrony is low. As the distance to the control line increases, this probability becomes higher. Just as an order parameter describes the transition between a solid and a liquid phase, our index would reflect the transition between a synchronous and a dyssynchronous phase of ventricular electrical conduction. The automatic analysis of a large ECG database of almost 2000 patients with a wide spectrum of ventricular conduction disorders allowed us to confirm that higher index values are found among patients with conduction abnormalities typically associated with dyssynchrony. Notably, the only paced patient with a dyssynchrony index close to control population values was a CRT user. These results led us to speculate that a clinically relevant threshold value for the dyssynchrony index would lie near 0.588, since this value adequately discriminates patients with LBBB or PM from the rest of the population (Figs. S4 and S5). Interestingly, some individuals with RBBB + LAFB and ILBBB approach this limit, suggesting that these patients could be potential CRT responders, provided that they fulfil the rest of the indication criteria.

While inappropriate patient selection may account for a subgroup of CRT non-responders, another subgroup could be explained by sub-optimal catheter placement or device configuration. Regarding the latter, an appropriate device configuration can result in synchrony improvement, even if LV catheter implantation is not optimal^30^. The optimization of CRT configuration can be attempted by modifying atrio-ventricular activation time (AV synchrony), ventricular electrode positioning and ventricular activation time (intra and interventricular synchrony), depending on the device and electrode type. Much effort has been invested in developing tools and methods for establishing optimal device parameters. During the implantation, it is common to systematically place the LV lead in the posterolateral or lateral branch of the coronary sinus vein, since this region most frequently accounts for delayed activation. However, even in patients with LBBB, the posterolateral zone is not always the most dyssynchronous region^31^. There is evidence supporting the use of pre-procedure echocardiography to improve CRT outcomes by using speckle-tracking to identify the last LV region to be activated. However, the use of this technique in real-world scenarios is scarce since it is extremely time-consuming and due to lack of availability and expertise, among other causes^32,33^. In the operating room, intra procedure image-guided synchrony assessment is even more technically difficult. The electrically-guided placement of LV leads in the last site to be activated is a more feasible alternative that is being currently evaluated in clinical trials, although there is still no concluding evidence of a beneficial effect^34,35^. Regarding post-implant optimization, the same limitations described for pre-procedure evaluation also hold true. Implementation difficulties and lack of strong evidence of clinical benefit preclude post-implant optimization from forming part of routine practice^36^. These difficulties are even greater when using multipolar electrodes, since there are even more possible stimulation sequences available. In this scenario, we believe that a patient-specific and real-time approach would be crucial for device optimization.

Our results show that the dyssynchrony index proposed in this study is able to discriminate between different alterations in the ventricular depolarization sequence. Interestingly, patients with low (high) index values also had low (high) TPS-SD values, which is a proxy for mechanical dyssynchrony (Fig. 4). This suggests that the dyssynchrony index could be potentially helpful in identifying mechanically dyssynchronous patients and in selecting stimulation locations and sequences during implant and post-implant optimization. Noteworthy, the index can be obtained in an easy, reproducible and operator-independent fashion. More echocardiographic studies, together with hemodynamic estimations of mechanical dyssynchrony, will be included in future clinical studies to further validate our computational pipeline.

This study sets the basis for the potential use of a ventricular synchrony quantification tool in the scenario of CRT. As a limitation, although an important number of ECGs were analysed, only a small amount of patients actually had a resynchronization device: one from the local BH database and 10 in the echocardiography cohort (6 conventional CRT devices and 4 His bundle/RV pacing devices). Hence, testing our computational pipeline in a larger population of patients with CRT devices will be key to further evaluate the method’s efficacy.

The dyssynchrony index implemented in our computational pipeline has proven to be a promising tool for ventricular electrical synchrony evaluation, with potential applications within the framework of CRT during candidate patient selection, device implant, and post-implant optimization. Whether this index correlates with other mechanical and clinical outcomes will be the matter of future studies.

## Methods

### Study design

**Figure 5:**
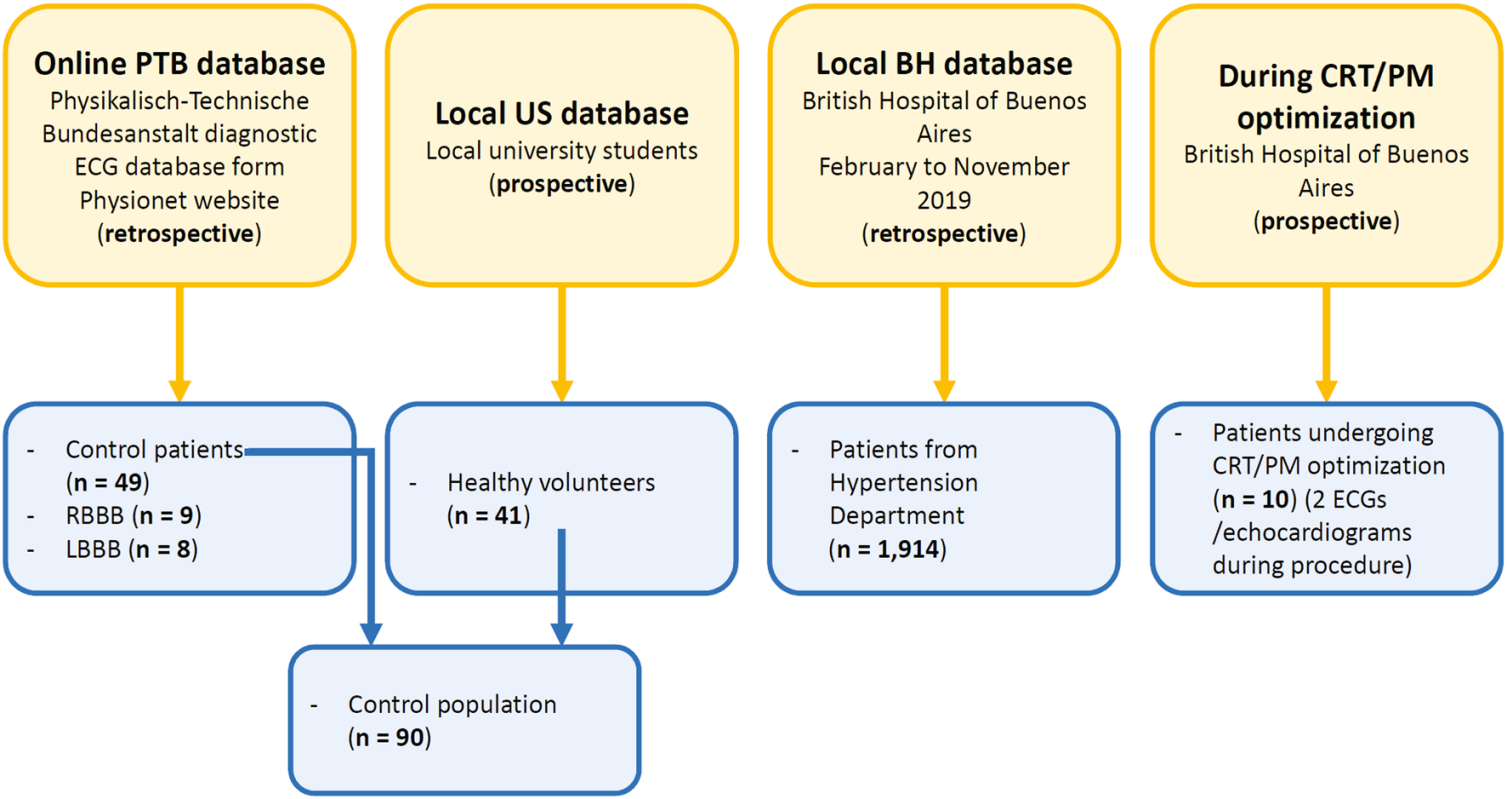
Sources of ECG recordings. CRT: cardiac resynchronization therapy. *PM* pacemaker, *RBBB* right bundle branch block, *LBBB* left bundle branch block.

This study was designed to test the ability of our newly developed dyssynchrony index to identify patients with impaired ventricular activation sequence, and to quantify the degree of this alteration. The study was an observational cross-sectional study that included multiple sources of ECGs (Fig. 5): an online database and a local database which were retrospectively collected, and a local university students database (local US database) which was prospectively collected. There were 10 additional patients for which prospective repeated measurements were obtained during CRT/PM device optimization. Briefly, ECGs from the online Physikalisch-Technische Bundesanstalt (PTB) database available on Physionet were used for pattern characterization (49 controls, 9 right bindle branch block—RBBB—, and 8 LBBB)^10,12^. The 49 controls from this database were included in a control population group, which also included the ECGs obtained prospectively from 41 local university students who were healthy volunteers with no history of cardiovascular pathology (local US database). ECGs from 1914 patients evaluated at the Hypertension Department of the British Hospital of Buenos Aires (Buenos Aires, Argentina) between February and November 2019 were retrospectively analysed (local BH database). ECG/echocardiogram recordings were obtained from 10 patients with PM or CRT devices during device optimization (20 ECGs in all, with 2 recordings from each patient). Patient characteristics of this last group are described in Table 1. ECGs with inacceptable signal-to-noise ratio or with extrasystolic beats were excluded (7% of all patients, see section titled “Method for detection of ventricular depolarization limits” for specific criteria).

### ECG equipment and software

12-lead ECG data were obtained using a digital electrocardiograph (ECG View beat, Eccosur, Buenos Aires, Argentina) connected to a personal computer with an acquisition software (ECGView V2.12.0.0, Eccosur, Buenos Aires, Argentina). Data sampled at 1000 Hz were stored as a “comma separated value” text file. Data analysis was executed by a custom software developed in MATLAB (MATLAB 2019a, MathWorks, Natick, MA, USA).

### General method for vectorcardiogram reconstruction and analysis

ECG recordings can be considered as 12 projections of a series of vectors that describe the electrical activity of the heart. The spatiotemporal trajectory of these vectors is denominated VCG. In this study, we developed a method to automatically extract VCGs from digitalized ECG signals. Briefly, through peak detection, polynomial fitting and numerical derivation, our algorithm detects a patient-specific voltage threshold. After processing, ventricular depolarization signal onset and stop were robustly detected (see next section, “Detection of ventricular depolarization limits”) to finally obtain the patients’ VCG. Detailed description of each step is given below. Pre-filtered (skeletal muscle and current line noise) data from the ECG machine were further filtered through a Butterworth filter. Drift and baseline wander correction was performed by subtracting a polynomial fit (10th order) to the original signal. Instantaneous values of the vector’s magnitude in the orthogonal planes (X, Y, Z) were obtained by the transformation matrix method described by Kors^11^. The instantaneous vector magnitude (*voltage*(*t*)) at time *t* was calculated as:

$$voltage\left( t \right) = \sqrt {\left( {X\left( t \right)} \right)^{2} + \left( {Y\left( t \right)} \right)^{2} + \left( {Z\left( t \right)} \right)^{2} }$$

Spatial vector speed (*speed*(*t*)) at time *t* was calculated as:

$$speed\left( t \right) = \sqrt {\left( {\frac{dX}{{dt}}\left( t \right)} \right)^{2} + \left( {\frac{dY}{{dt}}\left( t \right)} \right)^{2} + \left( {\frac{dZ}{{dt}}\left( t \right)} \right)^{2} }$$

### Detection of ventricular depolarization limits

**Figure 6:**
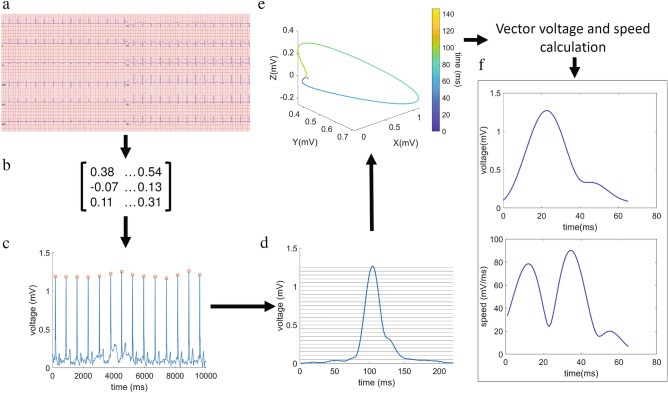
ECG-derived VCG extraction algorithm and calculation of instantaneous vector voltage and speed kinetics. (**a**) Raw ECG data is loaded into the program as input. (**b**) Kors transformation matrix is applied to the raw data. (**c**) Peaks are identified along the voltage (spatial vector magnitude) over time signal. (**d**) Patient mean depolarization signal (peak-aligned) limits are identified by a custom adaptive threshold method. (**e**) VCG extraction (colour bar representing time in ms). (**f**) Instantaneous voltage and speed signals over time are obtained. The figure was created using MATLAB R2019b (https://www.mathworks.com/).

A flow chart of the analysis procedure is depicted in Fig. 6. A 10-s recording of the 12-lead ECG for each patient was used. Kors’ transformation matrix was applied to the entire signal in order to obtain a single voltage-over-time recording. A 220-ms window surrounding each vector’s voltage signal peak was automatically detected (Fig. 6c). Since the signal before and after the ventricular depolarization (P-R and S-T segments, respectively) must be represented as an isoelectric segment in every ECG lead, the first and last points of this 220-ms time window were normalized to zero. These points are not necessarily isoelectric though, from a theoretical point of view, in most conditions there is no significant instantaneous resultant vector that could be sensed from the body surface. The depolarization signals (initially with a 220-ms length) were aligned using the peak of the vector’s voltage as reference. Ventricular depolarization signals were then averaged obtaining the average vector’s voltage signal (Fig. 6d) representative of all the complexes included in the 10-s recording. To establish the limits of the ventricular depolarization signal, an increasing threshold was applied to the patient’s average vector’s voltage signal in 0.001 mV steps (Fig. S6a). The signal length was evaluated in each iteration and a function describing the relation between the threshold and the signal length was obtained by fitting data to a 7th order polynomial function (Fig. S6b). The final patient-specific threshold was determined by finding the point where the normalized 1st derivative of this function reached − 0.275 ms/mV, being this last value established by trial-and-error (Fig. S6b,c). Finally, from the processed data we derived the patient’s average VCG signal as described in the previous section (Fig. S6d–f). The VCG obtained through this process is representative of all the ventricular depolarization signals included in the 10-s period. This method was able to found signal’s limits near the beginning and end of the depolarization (Fig. S1) and the dyssynchrony index (see next section, “Electrical synchrony assessment”) was found to be almost insensitive to changes in signal limits over a wide range (Fig. S7). Both the automatic signal limits identification and the insensitivity of dyssynchrony index to changes in those limits were tested over different conduction patterns (Normal conduction, RBBB, LBBB). ECGs with a low signal-to-noise ratio or with extrasystolic beats were excluded by imposing a threshold of 0.9 for the intra-patient ventricular depolarization signal correlation (Pearson’s correlation coefficient). As a consequence of this constrain, 7% of all ECGs analysed were excluded and the final global intra-patient signal correlation was 0.996 (ranging from 0.901 to 0.999).

### Electrical synchrony assessment

As mentioned in the Results and Discussion sections, we found a linear correlation between the speed time integral (STI) and the voltage time integral (VTI) in the control population. We defined the dyssynchrony index as the shortest distance between the patient’s VTI-STI coordinates and the line obtained from the control population’s linear fit. Since the magnitudes on the abscissa and ordinate were different, we first normalized each unit to arbitrarily selected values (100 mV s for VTI and 10,000 mV for STI) in order to nondimesionalize the data. Once the data were conveniently normalized, the dyssynchrony index of a patient with coordinates VTI and STI was calculated as:

$$dyssynchrony\ index = \frac{{\left| {k + m.VTI - STI} \right|}}{{\sqrt {1 + m^{2} } }}$$

where *k* and *m* are the intercept and the slope, respectively, obtained from the linear fit of the normalized control population data.

### Mechanical synchrony assessment (strain echocardiography)

All echocardiographic studies were performed with an iE33 echography system equipped with a 3-MHz sector transducer (Philips Medical Systems, Bothell, WA). Grayscale 2D cine loops were obtained at the standard apical views (4-chamber, 2-chamber, and apical long-axis). Mean frame rate was 61.6 ± 5.9 frames/s (mean ± SD). Mechanical dyssynchrony was assessed as the standard deviation of the time to minimum systolic strain (Time to Peak Strain Standard Deviation, TPS-SD)^17,18^. Briefly, DICOM images were imported to a commercially available software (Qlab 13.0, Philips Andover, MA) which automatically performed LV segmentation into 18 segments (6 segments for each region: basal, middle and apical) and endocardial surface detection. Time-strain data for all segments were exported to a commercially available spreadsheet program. Time to peak strain (starting from the onset of the QRS complex) for each of the 18 segments was detected to finally calculate TPS-SD.

### Statistical methods

Differences among groups were evaluated by a Wilcoxon rank sum test, since data were non-normally distributed. In all cases, tests were two-sided and considered a 0.05 level of significance and 80% power. Sample sizes (n values) were specifically stated in figures legends. The normality of data was evaluated by the Kolmogorov–Smirnov test. For establishing the cut-off value of the dyssynchrony index, a logistic regression analysis was performed and the dyssynchrony index value with the best discrimination ability between synchronous and dyssynchronous groups was selected using the highest Youden index^16^:

$$Youden\,index = sensitivity+specificity-1$$

Since there is a range of dyssynchrony indexes with the highest Youden index (0.52–0.65, Fig. S6), the mean was chosen as the final cut-off value (0.58). All statistical analyses were performed using MATLAB statistics toolbox (2019a) or Python’s SciPy package (Ver. 1.6.0).

### Ethics statement

The British Hospital of Buenos Aires’ institutional review board approved this study with a waiver of consent for ECGs retrospectively analysed, and with written informed consent required for ECGs/echocardiograms prospectively acquired during device optimization (protocol numbers 966 and 967). Informed consent was obtained from all participants prospectively enrolled. The study was conducted according to the principles of the Declaration of Helsinki (version 2013) and the International Conference on Harmonization Guidelines for Good Clinical Practice.

## Supplementary Information

Supplementary Figures.

## Data Availability

Sample ECG recordings for each typical conduction pattern can be obtained from PTB database at the following link: https://www.physionet.org/content/ptbdb/1.0.0/. The MATLAB/Python implementation of the analysis described in this work may be requested to the corresponding author through material transfer agreement or licensing.
