# Supplementary material for: Vectorcardiography-derived index allows a robust quantification of ventricular electrical synchrony
Source: Sci Rep. 2022 Jun 15;12:9961. doi: 10.1038/s41598-022-14000-8 (PMC9200867; doi:10.1038/s41598-022-14000-8)
Supplement: Supplementary file 1 — Supplementary Figures. [file 41598_2022_14000_MOESM1_ESM.docx]

**Supplementary information for:**

**Vectorcardiography-derived index allows a robust quantification of ventricular electrical synchrony**

Juan M. Fernández^1^ Ph.D., Damián N. Spagnuolo^1^ M.D., María T. Politi^2^ M.D., Iván Tello Santa Cruz^3^ M.D., Miguel Schiavone^3^ M.D., César Cáceres Monié^3^ M.D., Horacio A. Avaca^3^ M.D., Osvaldo Chara Ph.D.*^1,4,5^

^1^Systems Biology Group (SysBio), Institute of Physics of Liquids and Biological Systems (IFLySIB), National Scientific and Technical Research Council (CONICET), University of La Plata; La Plata, Argentina.

^2^Laboratory of Biomembranes, Institute of Physiology and Biophysics Bernardo Houssay (IFIBIO Houssay), School of Medicine, University of Buenos Aires; Buenos Aires, Argentina.

^3^Cardiology Department, British Hospital of Buenos Aires; Buenos Aires, Argentina.

^4^Center for Information Services and High Performance Computing, Technische Universität Dresden; Dresden, Germany.

5Instituto de Tecnología, Universidad Argentina de la Empresa (UADE), Buenos Aires, Argentina.

*Corresponding author. Email: osvaldo.chara@tu-dresden.de

**Content:**

[Figure S1](#S1). Representative plots of vectorcardiogram (VCG), vector voltage and speed for typical conduction patterns.

[Figure S2](#S2). Signal kinetics and values from the PTB database and from the US database.

[Figure S3](#S3). Dyssynchrony index calculated for patients from the Physikalisch-Technische Bundesanstalt (PTB) database and university students (US) database.

[Figure S4](#S4). Distribution of the dyssynchrony index in different conduction patterns from the British Hospital (BH) database.

[Figure S5](#S5). Determination of the threshold of the dyssynchrony index.

[Figure S6](#S6). Signal limits detection and vectorcardiogram (VCG) extraction.

[Figure S7](#S7). Evaluation of the sensitivity of the dyssynchrony index to the selection of signal limits.

**Figure S1. Representative plots of vectorcardiogram (VCG), vector voltage and speed for typical conduction patterns.**

Representative VCGs from patients with normal conduction (a), RBBB (d) and LBBB (g) and their corresponding voltage (b, e, h) and speed (c, f, i) time courses. LBBB signals show greater vector magnitudes with slower movements in the vector field as compared with controls, while RBBB signals show intermediate values for vector magnitudes and speeds that are between LBBB and control values. The VCGs were extracted from the digitalized ECGs signals available at the online PTB database (See the average voltage and speed time courses from the entire population of that database in Fig. 2). The figure was created using MATLAB R2019b (<https://www.mathworks.com/>).

**Figure S2. Signal kinetics and values from the PTB database (n = 49) and from the US database (n = 41)**

(a) Spatial vector magnitude (voltage) over time for control subjects from PTB database (blue) and from University Students database (US, red). (b) Vector speed over time for control subjects from PTB database (blue) and from US database (red). (c) Voltage time integral (VTI) for every control patient from PTB database (blue diamonds) and from US database (red circles). Blue and red continuous lines represent global median for each population with its respective 25^th^ – 75^th^ percentiles (discontinuous lines). (d) Speed time integral (STI) for every control patient from PTB database (blue diamonds) and from US database (red circles). Blue and red continuous lines represent global median for each population with its respective 25^th^ – 75^th^ percentiles (discontinuous lines). The figure was created using Python’s Matplotlib library (v.3.4.2.)^37^.

**Figure S3. Dyssynchrony index calculated for patients from the Physikalisch-Technische Bundesanstalt (PTB) database and university students (US) database.**

(a) Control voltage time integral (VTI) and speed time integral (STI) values from the PTB and US database (blue dots, n = 90) were used to obtain the control population fit (blue continuous line). Dashed lines represent 95% confidence intervals (CI), colorbar represents the dyssynchrony index as defined in the Methods section. (b) Right bundle branch block (RBBB) patients from the PTB database (n = 9) with control population fit and CI. (c) Left bundle branch block (LBBB) patients from the PTB database (n = 8) with control population fit and CI. (d) Frequency distribution of the dyssynchrony index for each conduction pattern, showing lack of overlap between LBBB and the other groups. The figure was created using MATLAB R2019b (<https://www.mathworks.com/>).

**Figure S4. Distribution of the dyssynchrony index in different conduction patterns from the British Hospital (BH) database.**

Violin plot showing the distributions, median and interquartile range of each conduction pattern found over 1,914 ECGs from the HB database. Some patients with RBBB+LAFB or ILBBB showed a dyssynchrony index close to LBBB and PM group values. Notably, the only CRT patient presents a dyssynchrony index much lower than PM group values. NC: normal conduction. IRBBB: incomplete right bundle branch block. LAFB: left anterior fascicular block. LPFB: left posterior fascicular block. RBBB: right bundle branch block. RBBB+LAFB: right bundle branch block and left anterior fascicular block. ILBBB: incomplete left bundle branch block. LBBB: left bundle branch block. PM: pacemaker. CRT: cardiac resynchronization therapy. The figure was created using MATLAB R2019b (<https://www.mathworks.com/>).

**Figure S5. Determination of the threshold of the dyssynchrony index.**


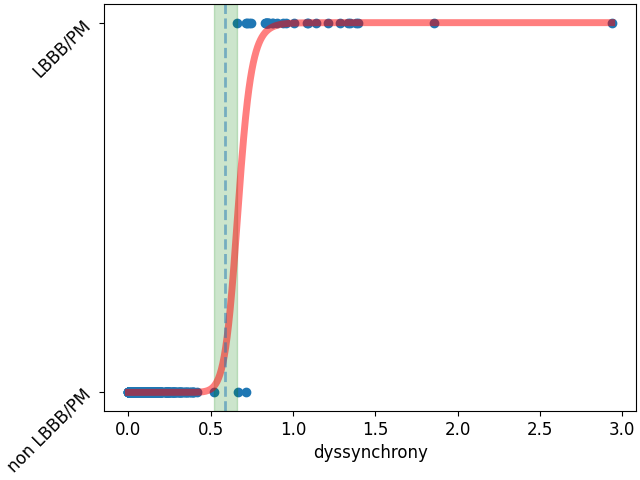


ECGs from the Physikalisch-Technische Bundesanstalt (PTB) database, control ECG from university students (US) database and the British Hospital (BH) database (n = 2,021, blue dots) were grouped into LBBB/PM or non LBBB/PM groups. By performing a logistic regression, a probability function was obtained (red line) and a cut-off of the dyssynchrony index separating both groups was identified by finding the higher values for the Youden index (ranging from 0.52 to 0.65, green shaded area), whose average, 0.58, is depicted (dashed vertical line). LBBB: left bundle branch block. PM: pacemaker. The figure was created using Python’s Matplotlib library (v.3.4.2.)^37^.

**Figure S6. Signal limits detection and vectorcardiogram (VCG) extraction. Normal conduction.**

(a) Patient mean depolarization signal (peak-aligned). An increasing threshold was applied with 0.001 mV steps, evaluating signal length above the threshold in every iteration. (b) Length-threshold data (orange) was fitted to a polynomial function (blue) and then its normalized first derivative (c) was used to select the final threshold (normalized 1^st^ derivative = -0.275 ms/mV, defined by trial-and-error). Representative vectorcardiogram (d), spatial vector magnitude (voltage, e) and vector speed (f) for normal conduction. Colorbar in panel d represents time (ms). The figure was created using MATLAB R2019b (<https://www.mathworks.com/>).

**Figure S7: Evaluation of the sensibility of the dyssynchrony index to the selection of signal limits.**

Voltage over time (a) and speed over time (b) plots of a representative patient with normal conduction (control). Colored dots represent the signal’s limits in each iteration. Dyssyncronny index values calculated for different signal limits in the control population (university students (US) database and controls from online Physikalisch-Technische Bundesanstalt (PTB) database, n = 90) (c): through an iterative process, the signals tails were symmetrically reduced by subtracting 0 to 20 ms from each side in 1 ms steps (colored dots) and the dyssynchrony index was calculated on each iteration. Panels (d) to (f) and (g) to (i) show equivalent results for right bundle branch block (RBBB) and left bundle branch block (LBBB), respectively (both from PTB database). The dyssynchrony index calculated for each iteration was almost insensitive to changes in the points selected as the beginning and end of the ventricular depolarization, independently of the conduction pattern. The figure was created using Python’s Matplotlib library (v.3.4.2.)^37^.
